# ﻿A revision of the *Mallomonas guttata* species complex (Synurales, Chrysophyceae) based on morphological and molecular criteria

**DOI:** 10.3897/phytokeys.268.178310

**Published:** 2025-12-22

**Authors:** Evgeniy S. Gusev, Marina E. Ignatenko, Nikita A. Martynenko, Hoan Tran, Chris Rey Lituanas, Yulia A. Podunay

**Affiliations:** 1 Severtsov Institute of Ecology and Evolution, Russian Academy of Sciences, Leninsky Prospect 33, 119071 Moscow, Russia Severtsov Institute of Ecology and Evolution, Russian Academy of Sciences Moscow Russia; 2 Institute for Cellular and Intracellular Symbiosis, Orenburg Federal Research Center, Ural Branch, Russian Academy of Sciences, Pionerskaya Street, 11, 460000, Orenburg, Russia Institute for Cellular and Intracellular Symbiosis, Orenburg Federal Research Center, Ural Branch, Russian Academy of Sciences Orenburg Russia; 3 Joint Vietnam-Russia Tropical Science and Technology Research Center, 63 Nguyen Van Huyen, Nghia Do, Cau Giay, Hanoi, Vietnam Joint Vietnam-Russia Tropical Science and Technology Research Center Hanoi Vietnam; 4 Central Mindanao University, Maramag, Bukidnon, Mindanao, Philippines Central MIndanao University Maramag Philippines; 5 T.I. Vyazemsky Karadag Scientific Station, Natural Reserve of the Russian Academy of Sciences, Nauki street, 24, 298188, Kurortnoe, Feodosiya, Russia T.I. Vyazemsky Karadag Scientific Station, Natural Reserve of the Russian Academy of Sciences Feodosiya Russia

**Keywords:** *
Mallomonas
*, morphology, new species, phylogenetic analysis, species complex, tropics

## Abstract

*Mallomonas guttata* species complex was investigated in a tropical region using electron microscopy and molecular phylogeny. A study of six algal cultures belonging to the *M. guttata* morphotype revealed two main clades based on two datasets (SSU rDNA+*rbc*L cpDNA, and ITS rDNA). This work establishes the phylogenetic position of *M. guttata* for the first time. We propose the new species, *M. monilifera***sp. nov.**, for organisms from the second clade, which were distinguished by the presence of a rim around the pits located on the shield on their scales and showed considerable genetic distances from the *M. guttata* clade. Within this new species, the strains formed two subclades, for which we propose two subspecies. Scales of the new species were also found in several water bodies on Mindanao Island (Philippines).

## ﻿Introduction

The genus *Mallomonas* Perty was described in 1852 (Perty 1852), and since then, numerous studies have been devoted to investigating its flora and systematics in various regions of the world (Kristiansen and Preisig 2007). The modern systematics of the genus is based on electron microscopy (EM) studies initiated in the 1950s, which led to a qualitative leap in the development of the species concept (Škaloud et al. 2013). Currently, about 250 taxa have been described using EM (Siver 2024). Species identification is based on the ultrastructure of the silica scales and, to a lesser extent, the bristles that cover the cells of *Mallomonas* (Asmund and Kristiansen 1986; Siver 1991). Based on scale ultrastructure, 19 main sections within the genus have been delineated (Kristiansen and Preisig 2007).

Recently, molecular methods have been increasingly applied in the study of the genus. Initial works made it possible to construct a general phylogeny of the genus and showed that, in most cases, the major clades correspond to the sections distinguished based on morphological structure (Jo et al. 2011, 2013; Siver et al. 2015). Several recent studies have proposed the recognition of two additional sections based on molecular data (Hao et al. 2024; Knotek et al. 2025). However, as molecular data have accumulated, it has been revealed that many morphotypes, especially those considered widespread, often represent complexes of closely related taxa. This has led to a re-evaluation of the taxonomic significance of various characters, and to the revision and description of new species of *Mallomonas* from different sections (Jo et al. 2013; Kim et al. 2014; Jeong et al. 2019; Gusev et al. 2024a, b, c, d, e, 2025a; Martynenko et al. 2024; Knotek et al. 2025; Podunay et al. 2025). Most sections of *Mallomonas* have not yet been sufficiently studied using molecular methods, and for some sections, such data are entirely lacking (Siver et al. 2015; Čertnerová et al. 2019).

The section Papillosae Asmund & Kristiansen is one of those for which molecular data are available for only a small number of species. The representatives of this section are characterized by the presence of papillae on the shield and a dome on all scale types. The main diagnostic features are the presence or absence of anterior submarginal ribs, internal reticulation, presence or absence of ribs on the anterior flange, and the density of the papillae on the shield and their ornamentation (Kristiansen and Preisig 2007). Complexes of morphologically similar taxa that are genetically distinct have already been identified within the section. This includes the *Mallomonas kalinae* / *M. furtiva* species group, which differs in the density of papillae on the shield, the presence/absence of perforations on the base plate, and bristle morphology (Gusev et al. 2018). Recently, *M. laureana* Knotek & Škaloud was described, differing from *M. rasilis* Dürrschmidt by the presence of internal reticulation on the shield and internal struts on the V-ribs, dome morphology, and scale size (Knotek et al. 2025). Different genetic lineages have also been demonstrated for the *M. papillosa* Harris & Bradley morphotype (Škaloud et al. 2025b).

One of the widespread and variable species within the section is *Mallomonas guttata* Wujek, most frequently reported in water bodies of the tropical region (Kristiansen and Preisig 2007). Studies of freshwater habitats in Vietnam have shown that this species is common in various parts of the country (Gusev et al. 2023). During our research, we successfully isolated two morphotypes from this group into culture. The aim of this article is to conduct a revision of the *M. guttata* complex, describe a new species, and clarify the distribution data of these taxa.

## ﻿Materials and methods

Water samples from six localities in three provinces in Vietnam were used for culture isolation (Table 1). Additionally, fixed material from seven localities in two more provinces was analyzed (Table 2). Samples in Vietnam were taken during expeditions of the Joint Vietnam-Russia Tropical Science and Technology Research Center (the “Ecolan 3.2” project) in 2010–2022. Descriptions of the climatic and geographical features of the provinces are given in previously published papers (Gusev et al. 2017, 2019, 2021, 2022a; Tran and Mazei 2018; Tran et al. 2022). In general, this area has a tropical monsoon climate with high annual precipitation varying in timing and amount between provinces, and high relative humidity (Schmidt‐Thomé et al. 2015). Fixed material from six samples (five localities) collected in two provinces on Mindanao Island (Philippines) was studied (Table 2). This region is also characterized by a tropical monsoon climate.

**Table 1.** List of studied strains with information about localities in Vietnam and environmental parameters (Cond. – specific conductance, µS cm^-1^; T – temperature, °C; SSU rDNA, ITS rDNA, *rbc*L – GenBank accession numbers).

Strain	Locality	GPS	pH	Cond.	T	SSU+ITS	*rbc*L	Identification
Vietnam								
Dak Lak Province								
С6/9, (authentic)	Water pool near stream	12°49.772'N, 108°55.129'E	6.6	55	30	PX630187	PX637837	M. monilifera subsp. monilifera
Khanh Hoa Province								
C10/4	Swamp area near Cai River	12°15.494'N, 108°48.858'E	5.7	92	30	PX630188	PX637838	M. monilifera subsp. monilifera
Dong Nai Province								
39_18 (authentic)	Nuoi Thuong, unnamed water body, Cat Tien National Park	11°24.406'N, 107°24.388'E	7.0	35	36	PX630185	PX637835	M. monilifera subsp. dongnaiensis
32	Ta Lai reservoir, Cat Tien National Park	11°23.275'N, 107°21.062'E	6.1	56	30	PX630186	PX637836	M. monilifera subsp. dongnaiensis
CT201	Pool near swamp Bau Thai swamp, Cat Tien National Park	11°30.377'N, 107°21.658'E	5.2	72	31	PX630184	PX637834	M. monilifera subsp. dongnaiensis
D8/22	Bau Thai swamp, Cat Tien National Park	11°30.382'N, 107°21.616'E	5.5	77	30	PX630189	PX637839	*M. guttata*

**Table 2.** Basic characteristics of the localities studied by morphological approach (Cond. – specific conductance, µS cm^-1^; T – temperature, °C).

Provinces	Locality	GPS	Year	pH	Cond.	T	Identification
Vietnam							
Dong Nai	Bau Sau Lake, Cat Tien National Park	11°27.531'N, 107°20.700'E	2024	6.5	72	29	M. monilifera subsp. dongnaiensis
	Dac Lua swamp, Cat Tien National Park	11°31.018'N, 107°23.286'E	2024	5.7	8	29	M. monilifera subsp. dongnaiensis
	Nuoi Thuong, unnamed water body 2, Cat Tien National Park	11°24.407'N, 107°24.393'E	2024	6.6	32	29	M. monilifera subsp. dongnaiensis
	Nuoi Thuong, unnamed water body 1, Cat Tien National Park	11°24.432'N, 107°24.364'E	2024	6.7	24	30	*M. guttata*
Hue	Bau Sen Lake, Phong Dien District	16°34.173'N, 107°26.502'E	2019	5.0	37	30	M. monilifera subsp. monilifera
Danang	Pond in Hoi An city	15°52.684'N, 108°19.565'E	2024	7.0	380	32	M. monilifera subsp. monilifera
Khanh Hoa	Cam Ranh, pool in the sand	12°05.202'N, 109°11.000'E	2024	6.4	70	31	M. monilifera subsp. monilifera
Philippines							
Bukidnon	Lake Pinamaloy st. 1, Barangay Pinamaloy, Don Carlos	7°40.436'N, 125°00.063'E	2025	6.9	53	29	M. monilifera subsp. monilifera
	Lake Pinamaloy st. 2, Barangay Pinamaloy, Don Carlos	7°40.421'N, 125°00.141'E	2025	6.6	49	31	M. monilifera subsp. monilifera
	Opalon Stream, Barangay Butong, Quezon	7°47.777'N, 125°03.989'E	2025	7.1	200	27	M. monilifera subsp. monilifera,
	Opalon Swamp, Barangay Butong, Quezon	7°47.781'N, 125°03.979'E	2025	7.4	155	39	*M. guttata*
	Ricefield, Barangay Sampagar, Damulog	7°30.976'N, 124°57.421'E	2025	8.3	251	38	*M. guttata*
Misamis Oriental	Pond in Barangay Lunotan, Gingoog City	8°42.263'N, 125°00.923'E	2025	6.8	19	24	M. monilifera subsp. monilifera

Planktonic samples were collected using a plankton net with a 20 μm mesh size. Water mineralization and temperature measurements were performed using the Hanna device (HI 9828, Hanna Instruments, Inc., Woonsocket, RI, USA). Strains were isolated by Yu.A. Podunay in 2018. Cultures were deposited at the Collection of the Severtsov Institute of Ecology and Evolution, Russian Academy of Sciences (IEE RAS).

The study of the ultrastructure of scales and bristles of the *Mallomonas* species was carried out using scanning electron microscopy (SEM) on a Tescan Mira3 microscope (Tescan Brno, s.r.o, Brno, Czech Republic) at the Joint Usage Center «Instrumental methods in ecology» at the IEE RAS and at the Gagarin Center for the Identification and Support of Talented Children (Orenburgskaya oblast) and also using transmission electron microscopy (TEM) on a JEM-1011 transmission electron microscope in the Center of Electron Microscopy at Papanin Institute for Biology of Inland Waters, RAS. An aliquot of a sample applied to SEM stubs, dried at room temperature, and sputtered with gold using an ion-plasma sputtering system (Quorum Q150R ES plus; Quorum Technologies Ltd., London, UK). For studies with the transmission electron microscope (TEM), formvar-coated grids (EMS FF200-Cu-50, Electron Microscopy Sciences, Hatfield, PA, USA) were used.

Monoclonal strains were established by examination of micropipetted single cells under an inverted microscope. Non-axenic unialgal cultures were maintained in modified WC, DY-V and Waris-H liquid mediums (McFadden and Melkonian 1986, Andersen 2005) at 22 °C, in a growth chamber with a 12:12 h light:dark photoperiod with light intensity 50–100 µmol m^-2^s^-1^. Totally, six strains were isolated from different parts of Vietnam. They were used for further phylogenetic analysis for SSU rDNA + *rbc*L cpDNA and for ITS1-5.8S-ITS2 rDNA datasets.

The total DNA of the monoclonal culture was extracted using InstaGene^TM^ Matrix according to the manufacturer’s protocol. Fragments of the partial SSU rRNA (1717 bp) were amplified using the following pairs of primers: 18S-F (Katana et al. 2001) and 18L (Hamby et al. 1988). For ITS1-5.8S-ITS2 rRNA (548-554 bp) fragments, the pair of primers was used: KN1 (Wee et al. 2001) and Chryso_ITSR (Škaloud et al. 2012). Amplification of the *rbc*L cpDNA (654 bp) marker was performed using the primers *rbc*L_2F (Daugbjerg and Andersen 1997) and Synura_*rbc*LR (Gusev et al. 2018). Amplification of all studied fragments was carried out using the premade mix ScreenMix (Evrogen, Russia) for the polymerase chain reaction (PCR). The conditions of amplification for partial rDNA fragments were: an initial denaturation of 5 min at 95 °C, followed by 35 cycles at 94 °C for denaturation (30 s), 52 °C for annealing (30 s) and 72 °C for extension (40–90 s), and a final extension of 10 min at 72 °C. The conditions of amplification for the *rbc*L fragments were the same as for ribosomal fragments except for number of cycles (40) and annealing temperature (48 °C). The resulting amplicons were visualized by horizontal agarose gel electrophoresis (1.5%), colored with SYBR Safe (Life Technologies, Carlsbad, CA, USA). Purification of DNA fragments was performed with the ExoSAP-IT kit (Affymetrix, Santa Clara, CA, USA) according to the manufacturer’s protocol. All studied fragments were decoded from two sides using forward and reverse PCR primers and the Big Dye system (Applied Biosystems, Foster City, CA, USA), followed by electrophoresis using a Genetic Analyzer 3500 sequencer (Applied Biosystems, Foster City, CA, USA). Additionally, fragments of SSU rDNA were sequenced using internal primers 18S-826F (Choi et al. 2013) and picoR2 (Belevich et al. 2015) to assemble and check resulted sequencies. Received sequences were checked manually and assembled after using MegaX (Kumar et al. 2018).

Newly determined sequences and GenBank sequences of 81 other *Mallomonas* strains were included in the alignment. Also, synurophycean *Synura americana* Kynclová & Škaloud in Škaloud et al., *S. mammillosa* E. Takahashi and *Neotessella lapponica* (Skuja) B.Y. Jo, J.I. Kim, W. Shin, P. Škaloud & P.A. Siver were added to the dataset as outgroup taxa. The sequences were aligned using either global SILVA alignment in the SINA v1.2.11 (Pruesse et al. 2012) for SSU rDNA, or MAFFT v7 with auto strategy (Katoh et al. 2019) for *rbc*L cpDNA fragments. We performed two separate phylogenetic analyses: one based on concatenated partial SSU rDNA + *rbc*L cpDNA fragments, and the other used ITS1-5.8S-ITS2 rDNA sequences. The resulted SSU rDNA + *rbc*L cpDNA dataset (2371 bp) was partitioned into different genetic regions and the most appropriate substitution model for each partition was estimated separately, using the Bayesian information criterion (BIC) in the jModelTest 2.1.10 (Darriba et al. 2012). As the most fit model was selected GTR + G + I for the SSU rDNA. For each codon position of the protein-coding *rbc*L cpDNA gene, the best model was also tested. The BIC-based model selection procedure selected the following models: GTR + G + I for the first codon position, JC + I for the second codon position, and GTR + G for the third position. A dataset of ITS1-5.8S-ITS2 rDNA sequences (585 bp) was assembled, comprising the studied strain and nine other strains from the section Papillosae (15 strains in total). The dataset was aligned using MAFFT v7 with the ‘auto’ strategy. The substitution model HKY + G was chosen by jModelTest 2.1.10 for this dataset. Bayesian Inference (BI) analysis was conducted with MrBayes-3.2.5 (Ronquist and Huelsenbeck 2003). Three “hot” and one “cold” Markov chains were run for 15 × 10^6^ cycles in two repetitions with the selection of each 100^th^ generated tree. Phylogenetic tree and posterior branching probabilities were obtained after discarding the first 25% to produce estimate parameter models of nucleotide substitutions and likelihood. For the SSU + *rbc*L and ITS datasets, maximum likelihood phylogeny (ML) was constructed using IQ-TREE2 (Chernomor et al. 2016; Minh et al. 2020) with the models and partitions, described above. Phylogenetic tree obtained with Bayesian Inference was used as start tree for ML and bootstrap analysis with 1,000 replicates was used. Viewing and editing of all phylogenetic trees were carried out in the programs FigTree (ver 1.4.2) and Adobe Photoshop CC (19.0).

## ﻿Results

**Figure 1.**
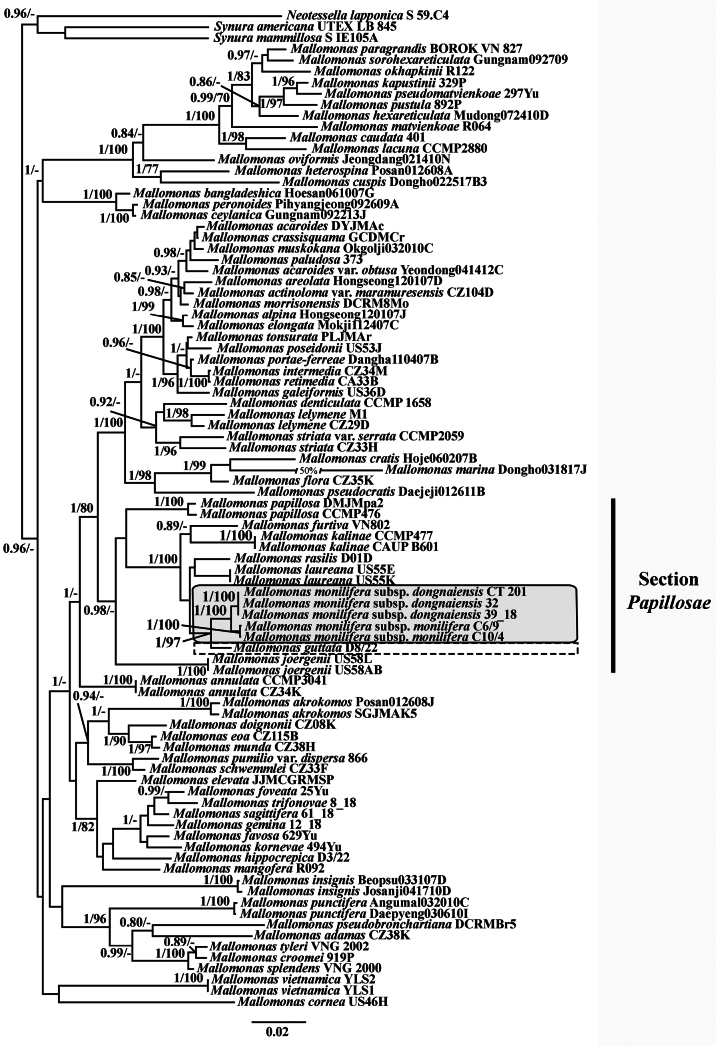
Bayesian consensus tree of the nuclear small subunit rDNA (SSU rDNA) and chloroplast *rbc*L concatenated data set. The Bayesian posterior probability (>0.80) and maximum likelihood bootstrap value (>70%) are shown left and right of the fraction line, respectively. Scale bar represents substitutions per site. New taxa, described in this research, are marked with boxes with solid lines, *Mallomonas guttata* – with dashed line rectangular.

**Figure 2.**
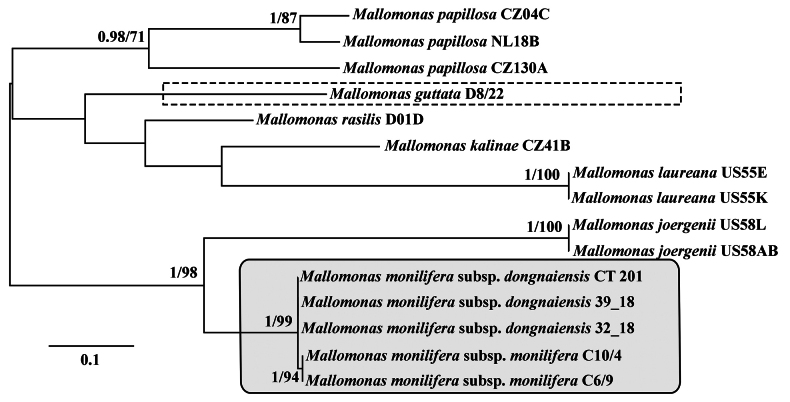
Unrooted Bayesian tree based on the ITS1-5.8S-ITS2 sequences of Mallomonas species from the section Papillosae. The Bayesian posterior probability (>0.80) and maximum likelihood bootstrap value (>70%) are shown left and right of the fraction line, respectively. Scale bar represents substitutions per site. New taxa, described in this research, are marked with the box with solid line, *Mallomonas guttata* – with dashed line rectangular.

In this work, six *Mallomonas* strains from Vietnam, morphologically similar to *M. guttata*, were studied. Molecular analysis inferred using maximum likelihood (ML) and Bayesian inference (BI) from combined dataset of nuclear encoded SSU rDNA and plastid-encoded *rbc*L showed that they were grouped into one large clade with the species *M. papillosa*, *M. rasilis*, *M. laureana*, *M. kalinae* Řezáčová, *M. furtiva* Gusev, Čertnerová, Škaloudová & Škaloud, and *M. joergenii* (Fig. 1). However, in this clade, strains D8/22, on the one side, and C6/9, C10/4, 39_18, 32_18, CT201 on the other, formed two separate phylogenetic lineages. The study of the ultrastructure of the scales also showed differences between the strains of the two new phylogenetic lineages. The morphology of strain D8/22 corresponds to the description of *M. guttata*, thus, we have determined the phylogenetic position of this species among members of the section Papillosae. The organisms from the second clade represent a species new to science. Moreover, two subclades are clearly distinguished within the second clade: C6/9, C10/4 and 39_18, 32_18, CT201. It is quite unexpected that the differences in ITS rDNA between these clades were smaller (p-distance = 0.007) than in *rbcL* (p-distance = 0.014). For the SSU rDNA region, the p-distance value was 0.001. Given that no clear morphological differences were identified (Table 3), but the strains form two subclades with small genetic distances (Figs 1, 2), we describe two subspecies within this group below.

**Table 3.** Morphometric characteristics of the strains and natural populations of *Mallomonas monilifera* sp. nov. and the strain of *Mallomonas guttata*.

Feature		Subspecies of *Mallomonas monilifera* sp. nov.		Natural populations of *Mallomonas monilifera* sp. nov.		*Mallomonas guttata* Wujek (strain D8/22)
		Mallomonas monilifera subsp. monilifera (strain C6/9)	Mallomonas monilifera subsp. dongnaiensis (strain 39_18)	Vietnam (subsp. dongnaiensis)	Philippines (subsp. monilifera)	
Body scales	size of scales, µm	3.5–4.5 × 2.0–2.8	4.1–5.0 × 2.2–2.9	4.8–5.6 × 2.7–3.3	3.8–4.5 × 2.5–2.8	3.2–4.1 × 1.9–2.4
	number of pits	7–27	9–34	22–32	5–18	6–18
Apical scales	size of scales, µm	2.8–3.4 × 2.0–2.3	3.5–4.0 × 2.4–2.6	3.3–4.7 × 2.2–3.0	3.7 × 2.8	2.5–2.8 × 1.8–2.1
	number of pits	1–8	3–14	9–26	12	3–13
Rear scales	size of scales, µm	2.6–3.2 × 1.6–2.1	3.2–3.6 × 1.5–2.1	n/a	n/a	2.1–2.8 × 1.6–1.9
	number of pits	1–14	6–17	n/a	n/a	2–5
Diameter of pits, µm		0.16–0.22	0.16–0.22	0.16–0.22	0.18–0.23	0.17–0.22
A visible rim around the pits		+	+	+	+	–
Length of bristles, µm		4.8–7.3	6.5–9.4	n/a	n/a	4.0–7.3

Thus, we provide an expanded description of *Mallomonas guttata* sensu stricto and describe a new species, comprising two subspecies, based on molecular and morphological data.

### ﻿ Mallomonas guttata

Taxon classificationPlantaeSynuralesMallomonadaceae

Wujek (strain D8/22 from Vietnam)

FA3CE181-12B9-5E66-9613-A8F6B7ABAADE

Figs 3, 4

#### Description.

Body scales are 3.2–4.1 × 1.9–2.4 μm, oval, tripartite, with a dome and a V-rib. The dome is completely or partially covered by papillae. Shield with densely and regularly spaced papillae arranged in distinct rows and 6–17 scattered circular pits. One rimmed base plate pore is situated in the proximal area of the shield lacking secondary siliceous layer at the base of the V-rib. The V-rib is conspicuous and rounded. Anterior flange is raised above the shield, covered with papillae. The posterior rim with a smooth surface, inner striation and encircles approximately half of the scale. Posterior flange is narrow and smooth. Apical scales are asymmetrical, 2.5–2.8 × 1.8–2.1 μm, possess a wing-like projection, and bear several circular pits on the shield. Rear scales are similar in basic structure to the body scales but smaller, 2.1–2.8 × 1.6–1.9 μm, and possess either a single or a few circular pits. Bristles are 4.0–7.3 μm smooth, curved, pointed.

**Figure 3.**
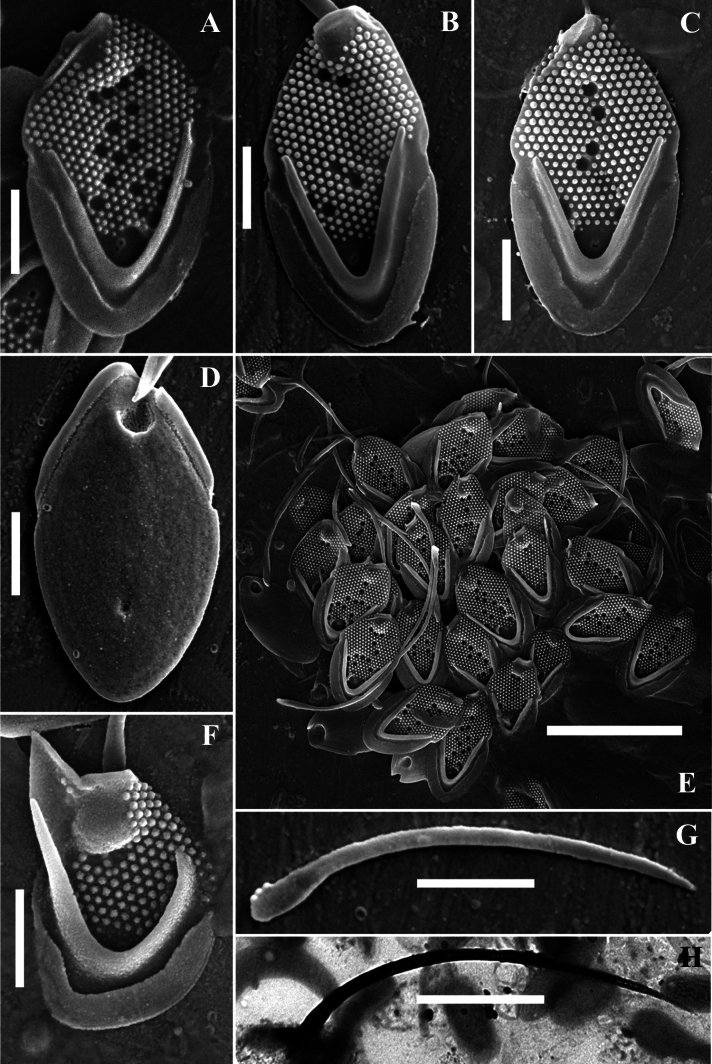
*Mallomonas guttata* Wujek *sensu stricto* (strain D8/22). **A–D.** Body scales; **D.** Body scale, undersurface view; **E.** Scales and bristles; **F.** Apical scale with wing-like projection; **G, H.** Bristles; **A–G.**SEM; **H.**TEM. Scale bars: 5 µm (**E**); 1 µm (**A–D, F–H**).

**Figure 4.**
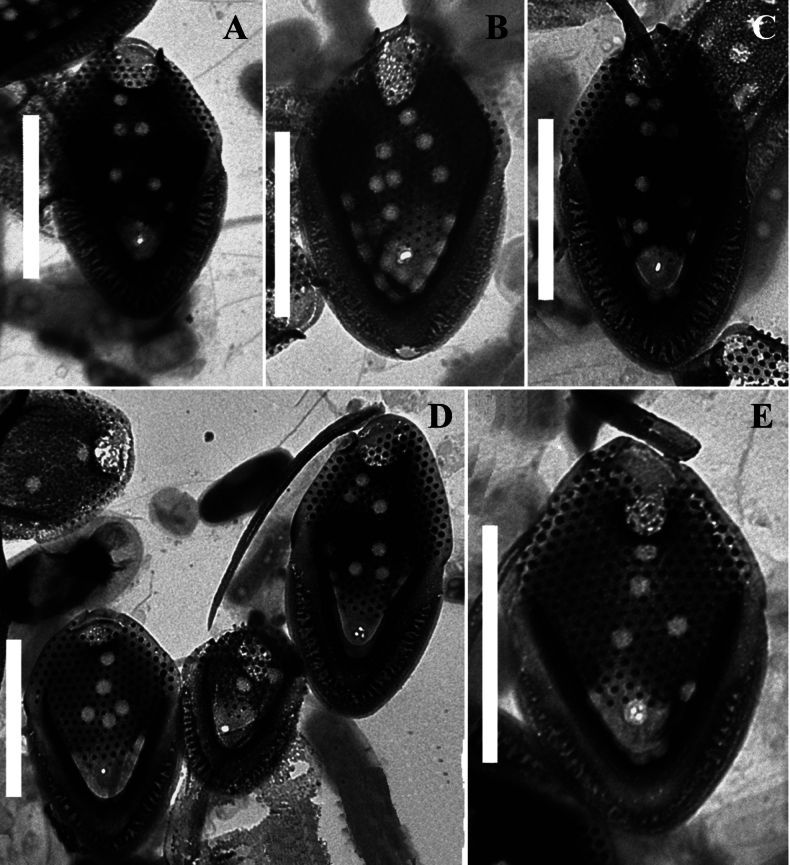
*Mallomonas guttata* Wujek *sensu stricto* (strain D8/22), TEM. **A–C, E.** Body scales; **D**. Body and rear scales. Scale bars: 2 µm.

### ﻿ Mallomonas monilifera

Taxon classificationPlantaeSynuralesMallomonadaceae

E.S.Gusev, Ignatenko, Martynenko & Podunay sp. nov.

B55F9FC1-D3DC-5539-9874-BB21D82579D4

#### Description.

Body scales 3.5–5.6 × 2.0–3.3 μm, oval, tripartite, with a dome and a V-rib. The dome is completely or partially is covered by papillae. Shield with densely and regularly spaced papillae arranged in distinct rows and circular pits. The number of pits varies from 5 to 34, usually there are more than 10. Each pit is surrounded by a thickened rim. The pits can be located in transverse rows or scattered across the shield. One rimmed base plate pore is situated in the proximal area of the shield lacking secondary siliceous layer at the base of the V-rib. The V-rib is conspicuous and rounded. Anterior flange is raised above the shield, covered with papillae. The posterior rim with a smooth surface, inner striation and encircles approximately half of the scale. Posterior flange is smooth. Apical scales are asymmetrical, 2.8–4.7 × 2.0–3.0 µm, can possess a wing-like projection, and bear several circular pits on the shield. Rear scales are similar in basic structure to the body scales but smaller, 2.6–3.6 × 1.5–2.1 µm, and possess several circular pits. Bristles are 4.8–9.4 µm, smooth, curved, pointed. Cysts were not observed.

#### Type.

**Figure 5.**
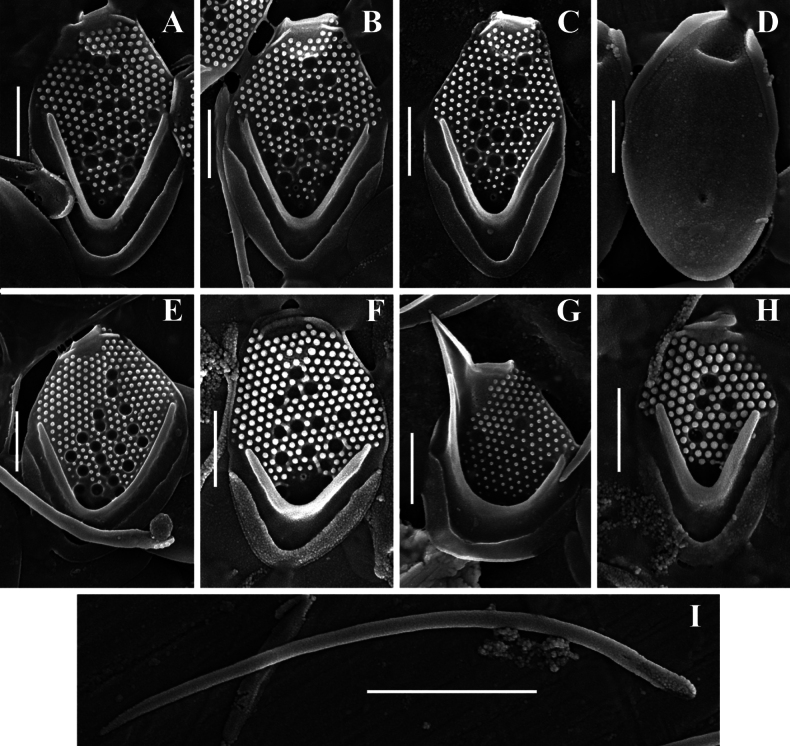
*Mallomonas monilifera* sp. nov.: M. monilifera subsp. monilifera subsp. nov. (strain C6/9), SEM. **A–D.** Body scales; **D.** Body scale, undersurface view; **E–G.** Apical scales; **G.** Apical scale with wing-like projection; **H.** Rear scale; **I.** Bristle. Scale bars: 2 µm (**I**); 1 µm (**A–H**).

Vietnam • Dak Lak Province, water pool near stream; 12°49.772'N, 108°55.129'E; 25 April 2018; Evgeniy S. Gusev leg. Holotype. Portion of a single gathering of cells on SEM stub 71_I_1 deposited at the Herbarium of the Steppe Institute of the Ural Branch of the Russian Academy of Sciences, Orenburg (ORIS). Fig. 5С is a representative scale from the type specimen.

#### Etymology.

The epithet “*monilifera*” reflects the characteristic arrangement of the shield pits, resembling a necklace.

### ﻿ Mallomonas monilifera subsp. monilifera

Taxon classificationPlantaeSynuralesMallomonadaceae

E.S.Gusev, Ignatenko, Martynenko & Podunay subsp. nov.

9D966A6A-2EBD-5154-8DF2-8616D17280AD

Figs 5, 6

#### Description.

Body scales 3.5–4.5 × 2.0–2.8 μm, oval, tripartite, with a dome and a V-rib. The dome is completely or partially is covered by papillae. Shield with densely and regularly spaced papillae arranged in distinct rows and circular pits. The number of pits varies from 7 to 27; usually there are more than 10. Each pit is surrounded by a thickened rim. The pits can be located in transverse rows or scattered across the shield. One rimmed base plate pore is situated in the proximal area of the shield lacking secondary siliceous layer at the base of the V-rib. The V-rib is conspicuous and rounded. Anterior flange is raised above the shield, covered with papillae. The posterior rim with a smooth surface, inner striation and encircles approximately half of the scale. Posterior flange is smooth. Apical scales are asymmetrical, 2.8–3.4 × 2.0–2.3 µm, can possess a wing-like projection, and bear several circular pits on the shield. Rear scales are similar in basic structure to the body scales but smaller, 2.6–3.2 × 1.6–2.1 µm, and possess several circular pits. Bristles are 4.8–7.3 µm, smooth, curved, pointed. Cysts were not observed.

#### Type.

Vietnam • Dak Lak Province, water pool near stream; 12°49.772'N, 108°55.129'E; 25 April 2018; Evgeniy S. Gusev leg. Holotype. Portion of a single gathering of cells on SEM stub 71_I_1 deposited at the Herbarium of the Steppe Institute of the Ural Branch of the Russian Academy of Sciences, Orenburg (ORIS). Fig. 5С is a representative scale from the type specimen.

**Figure 6.**
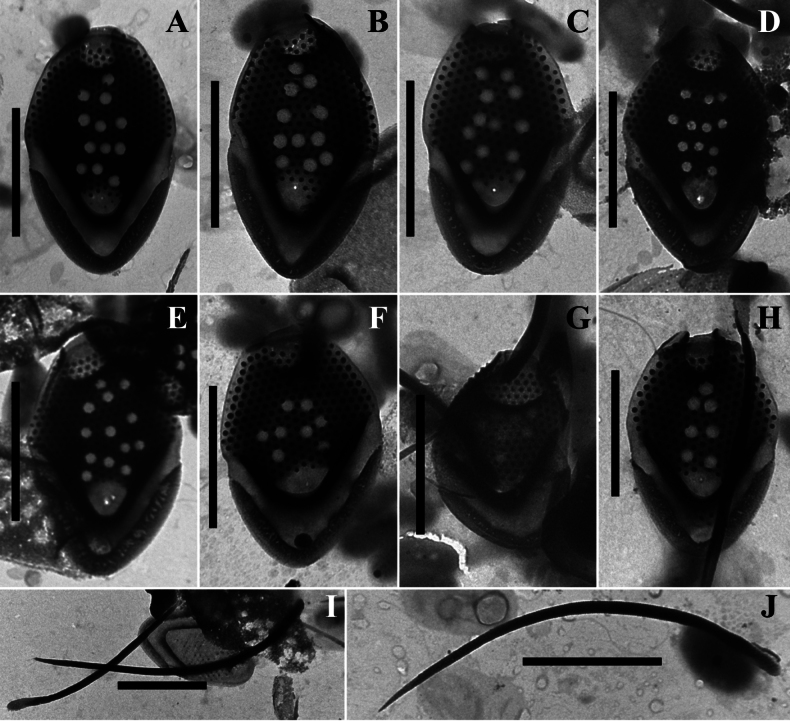
*Mallomonas monilifera* sp. nov.: M. monilifera subsp. monilifera subsp. nov. (strain C6/9), TEM. **A–E.** Body scales; **F, G.** Apical scales; **H.** Rear scale; **I, J.** Bristles. Scale bars: 2 µm.

#### Etymology.

The epithet “*monilifera*” reflects the characteristic arrangement of the shield pits, resembling a necklace.

#### Distribution and ecology.

In addition to the type locality, this subspecies has been observed in eight more localities in Vietnam and six localities in the Philippines (Tables 1, 2). Mallomonas monilifera subsp. monilifera subsp. nov. was found under the following environmental conditions: pH 5.0 to 7.1, specific conductance of 19 to 380 µS cm^-1^, a temperature range of 24–31 °C (Tables 1, 2).

### ﻿ Mallomonas monilifera subsp. dongnaiensis

Taxon classificationPlantaeSynuralesMallomonadaceae

E.S.Gusev, Ignatenko, Martynenko & Podunay subsp. nov.

440AAEE6-935A-515F-A000-03649A08979E

Fig. 7

#### Description.

Body scales 4.1–5.6 × 2.2–3.3 μm, oval, tripartite, with a dome and a V-rib. The dome is completely or partially covered by papillae. Shield with densely and regularly spaced papillae arranged in distinct rows and circular pits. The number of pits varies from 9 to 34, usually there are more than 10. Each pit is surrounded by a thickened rim. The pits can be located in transverse rows or scattered across the shield. One rimmed base plate pore is situated in the proximal area of the shield lacking secondary siliceous layer at the base of the V-rib. The V-rib is conspicuous and rounded. Anterior flange is raised above the shield, covered with papillae. The posterior rim with a smooth surface, inner striation and encircles approximately half of the scale. Posterior flange is smooth. Apical scales are asymmetrical, 3.3–4.7 × 2.2–3.0 µm, can possess a wing-like projection, and bear several circular pits on the shield. The rear scales are similar in structure to the body scales, but smaller, 3.2–3.6 × 1.5–2.1 µm, bear several circular pits. Bristles are 6.5–9.4 µm smooth, curved, pointed. Cysts were not observed.

#### Type.

**Figure 7.**
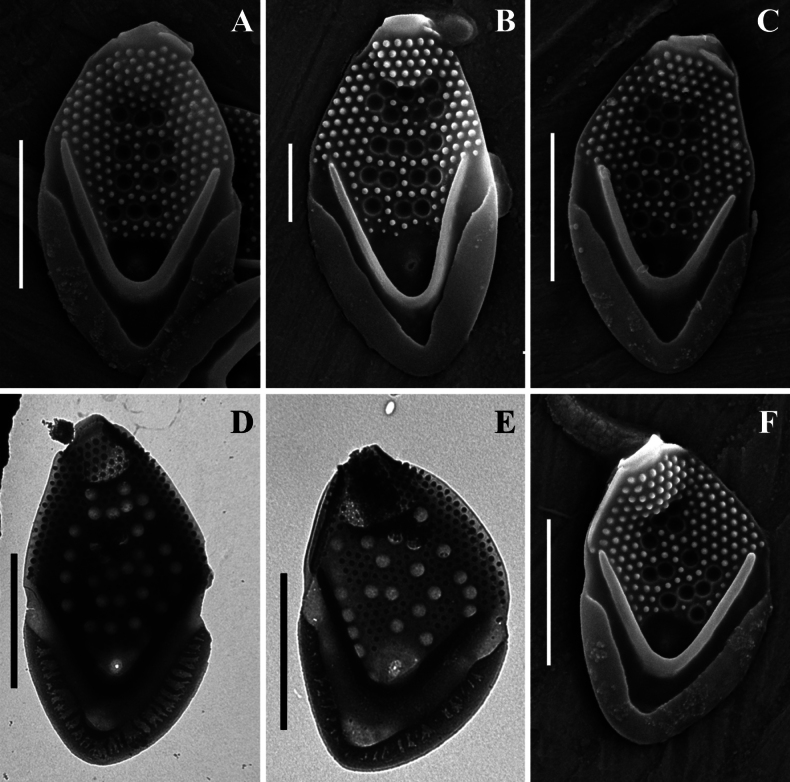
*Mallomonas monilifera* sp. nov.: M. monilifera subsp. dongnaiensis subsp. nov. (strain 39_18). **A–D.** Body scales; **E, F.** Apical scales; **A–C, F.**SEM; **D, E.**TEM. Scale bars: 2 µm (**A, C–F**); 1 µm (**B**).

Vietnam • Dong Nai Province, Cat Tien National Park, unnamed water body near Nuoi Thuong forest ranger station; 11°24.406'N, 107°24.388'E; 7 May 2018; Evgeniy S. Gusev leg. Holotype. Portion of a single gathering of cells on SEM stub 73_I_1 deposited at the Herbarium of the Steppe Institute of the Ural Branch of the Russian Academy of Sciences, Orenburg (ORIS). Fig. 7B is a representative scale from the type specimen.

#### Etymology.

The specific epithet “*dongnaiensis*” refers to Dong Nai Province, Vietnam, the area where the type material was collected.

#### Distribution and ecology.

In addition to the type locality, this subspecies has been observed in five more localities in Vietnam in Cat Tien National Park, Dong Nai Province (Tables 1, 2). Mallomonas monilifera subsp. dongnaiensis subsp. nov. was found under the following environmental conditions: pH 5.2 to 7.0, specific conductance of 8 to 72 µS cm^-1^, and a temperature range of 29–36 °C (Tables 1, 2).

## ﻿Discussion

*Mallomonas guttata* Wujek was described from a small lake in the province of Sarapiquí, Costa Rica in 1984 (Wujek 1984). According to the description, the species is characterized by elongated ellipsoidal cells, 7–12 × 3–6 µm. The scales are tripartite, 3–4 × 2–3 µm, possessing a dome, a V-rib, a posterior flange, and a posterior rim. The shield of the scale is covered with papillae arranged at equal distances from each other in straight longitudinal or oblique rows; the shield also has rounded depressions (Wujek 1984). Based on records from various countries, such as the USA (Wujek 1984; Wujek and Timpano 1984), Japan (Takahashi 1978), and Canada (Nicholls 1982), it was concluded that the species has a cosmopolitan distribution (Wujek 1984).

In 1985, a species morphologically similar to *Mallomonas guttata*, *M. perforata* Cronberg & Hickel, was described from Lake Bratan (Danau Bratan), Bali Island, Indonesia (Cronberg and Hickel 1985). The cells discovered by Cronberg and Hickel (1985) in Indonesia were larger than those found in Costa Rica (15.5 × 6.5 µm vs. 7–12 × 3–6 µm) but were covered with smaller scales (3.2 × 1.5 µm vs. 3–4 × 2–3 µm). Cronberg and Hickel (1985) also suggested a wide distribution for this species. Currently, *M. perforata* Cronberg & Hickel is considered a synonym of *M. guttata* (Kristiansen and Preisig 2007).

In 1989, while studying the flora of lakes in the central and southern part of the state of Michigan (USA), Wujek and Igoe (1989) discovered scales that differed from the type species *Mallomonas guttata* by a significantly smaller number of depressions (pits) on the shield surface. This led the authors to suggest the existence of a new species or a variety morphologically close to *M. guttata*. Notably, in the same year, M. guttata var. simplex K.H. Nicholls was described by Nicholls (1989) from a pond in the province of Ontario (Canada). This variety differs from M. guttata var. guttata in its shield ornamentation, specifically by having only a single row of large depressions (pits) located along the central longitudinal axis of the scale. This taxon is currently accepted, although it can be considered dubious, as it has been indicated that scales with a small number of pores arranged in a single row were found on the same cell as scales with a larger number of pores scattered across the shield (Gusev et al. 2017).

In subsequent years, scales identified as Mallomonas guttata var. guttata were recorded in various regions of the world, including Korea (Kristiansen et al. 1990), China (Kristiansen and Tong 1989; Wei and Yuan 2001), Madagascar Island (Hansen 1996), Papua New Guinea (Vyverman and Cronberg 1993), Ecuador (Wujek and Dziedzic 2005), the USA (Siver and Lott 2006), and Argentina (Siver 1997; Vigna and Siver 2003). For a long time, it was believed that the species had a wide but uneven distribution and occurred mainly in tropical and subtropical regions (Wei and Yuan 2001; Kristiansen and Preisig 2007). In 2012, *M. guttata* was discovered for the first time in Europe, specifically in the Aquitaine Region, France (Němcová et al. 2012). It was subsequently found in Austria (Pichrtová et al. 2013). Recent studies have also revealed *M. guttata* in the chrysophyte flora of Vietnam (Gusev et al. 2023), Philippines (Gusev et al. 2025b) and Australia (Nemcova and Diaz-Pulido 2023).

It should be noted that in some cases, scales discovered in various regions of the world differed from the type species in size or morphology. For example, scales measuring 5.3 × 3.2 µm were reported in China (see fig. 22, p. 176 in Wei and Yuan (2001)), and even larger scales were recorded in Ecuador — 5.9–6.3 × 3.8–3.9 µm (see fig. 6, p. 5 in Wujek and Dziedzic (2005)). In China, scales with a rim of smaller depressions (pits) compared to those on the shield were also observed; these depressions are located at the angle of the V-rib and surround the pore (see fig. 23, p. 176 in Wei and Yuan (2001)). Furthermore, many authors frequently reported scales whose dome was partially or completely covered with papillae (Kristiansen and Tong 1989; Kristiansen et al. 1990; Hansen 1996; Siver 1997), whereas according to the protologue, the scales of *Mallomonas guttata* have a smooth dome. These morphological differences from the type species, combined with the wide distribution of the taxon, provide grounds to suggest the existence of cryptic or pseudocryptic taxa and indicate the necessity for a thorough study of the species *M. guttata*.

Our study demonstrates that there are at least two genetically different morphotypes similar to *Mallomonas guttata*. One of the morphotypes corresponds to the description of *M. guttata*. However, it should be noted that the original description of *M. guttata* by D.E. Wujek (1984) is insufficiently detailed and contains several inaccuracies. In particular, the author identified only two scale types, interpreting scales with a wing-like projection as posterior scales (see fig. 4, p. 313 in Wujek (1984)). The description of the body scales lacks such significant diagnostic features as data on the number of depressions (pits), and the pattern of their arrangement on the shield. The dome is described as smooth, whereas the TEM images provided in the work clearly show papillae extending from the shield onto the scale dome (see figs 2–4, p. 313 in Wujek (1984)). Furthermore, the author only described the shape of the bristles, omitting data on their size.

Our data allowed for a more precise characterization of the scales and bristles of this taxon. Investigation of the Vietnamese isolate of *Mallomonas guttata* (D8/22) revealed three main scale types: apical scales with a wing-like projection, oval body scales, and rear scales, similar in morphology to body scales but smaller in size. The dimensions of the body scales in the isolate D8/22 are comparable to the description of *M. guttata* (3.2–4.1 × 1.9–2.4 µm vs. 3.0–4.0 × 2.0–3.0 µm, respectively). The dome of the scales in Vietnamese culture is partially or completely covered with papillae. The number of pits on the shield of the scales in isolate D8/22 varies from 6 to 18, compared to the 9–10 pits calculated from the images in the *M. guttata* description (see figs 2, 3, p. 313 in Wujek (1984)). The pits are scattered across the shield surface in both the Vietnamese isolate (Figs 3, 4) and the type of *M. guttata*. The bristles of the isolate D8/22 are smooth, which aligns with the description by D.E. Wujek (1984), who noted that the bristles are unserrated. Thus, it can be concluded that our study identified a morphotype corresponding to the description of *M. guttata*, and we provide its phylogenetic position. On the concatenated SSU rDNA-*rbcL* phylogenetic tree, the *M. guttata* strain (D8/22) clusters within a clade containing other representatives of the section Papillosae, most closely with *M. laureana*, *M. rasilis*, *M. kalinae*, and *M. furtiva*.

Given our discovery of new lineages within the *Mallomonas guttata* morphotype, it is appropriate to once again compare the data, taking into account our strain from Vietnam and *Mallomonas perforata* described from Indonesia (Cronberg and Hickel 1985) and later synonymized (Nicholls 1989). Based on our data on the ultrastructure of scales from the strain D8/22 of *M. guttata*, we believe that the previous synonymization of the taxa *M. perforata* and *M. guttata* was justified, even considering the insufficiently detailed original description of *M. guttata* by D.E. Wujek (1984). According to the description (Wujek 1984; Cronberg and Hickel 1985), the differences in scale size (3.2 × 1.5 µm in *M. perforata* vs. 3.0–4.0 × 2.0–3.0 µm in *M. guttata* and 3.2–4.1 × 1.9–2.4 µm for the strain D8/22) are minor. The scales of *M. perforata*, as in *M. guttata*, are oval, tripartite, with a dome and a V-rib. In both taxa, the dome is prominent, smooth or partially to completely covered with papillae. The shield is covered with papillae arranged in regular, dense rows and bears several circular pits. The V-rib is rounded. In the angle of the V-rib, a secondary siliceous layer is absent, and there is one rimmed base plate pore. The anterior flange is covered with papillae. The posterior rim has a smooth surface and inner striation. The posterior flange is narrow and smooth. The bristles of *M. perforata* and *M. guttata* also share identical morphology: they are smooth, curved, and pointed. Therefore, given the presence of genetically divergent lineages, a final resolution of this issue is only possible upon obtaining molecular data from the type locality of *M. perforata*.

*Mallomonas monilifera* forms a distinct clade on the phylogenetic tree, sufficiently distant from *M. guttata* (Fig. 1). *Mallomonas monilifera* forms two genetic lineages, for which we propose subspecies rank. We also observed variations in both scale size and the number of pits on the shield among different strains and natural specimens of *M. monilifera*. That is, two groups can also be distinguished based on morphological characters, although they are poorly differentiated from each other. In general, both subspecies of *M. monilifera* are characterized by larger scales compared to *M. guttata* (3.5–5.6 × 2.0–3.3 µm vs. 3.2–4.1 × 1.9–2.4 µm for strain D8/22) and higher number of pits (up to 34 pits in *M. monilifera* vs. 9–10 pits for *M. guttata* according to the description (Wujek 1984), and 6–17 for strain D8/22). However, the two subspecies of *M. monilifera* differ in both scale size and the number of pits on the shield surface.

**Figure 8.**
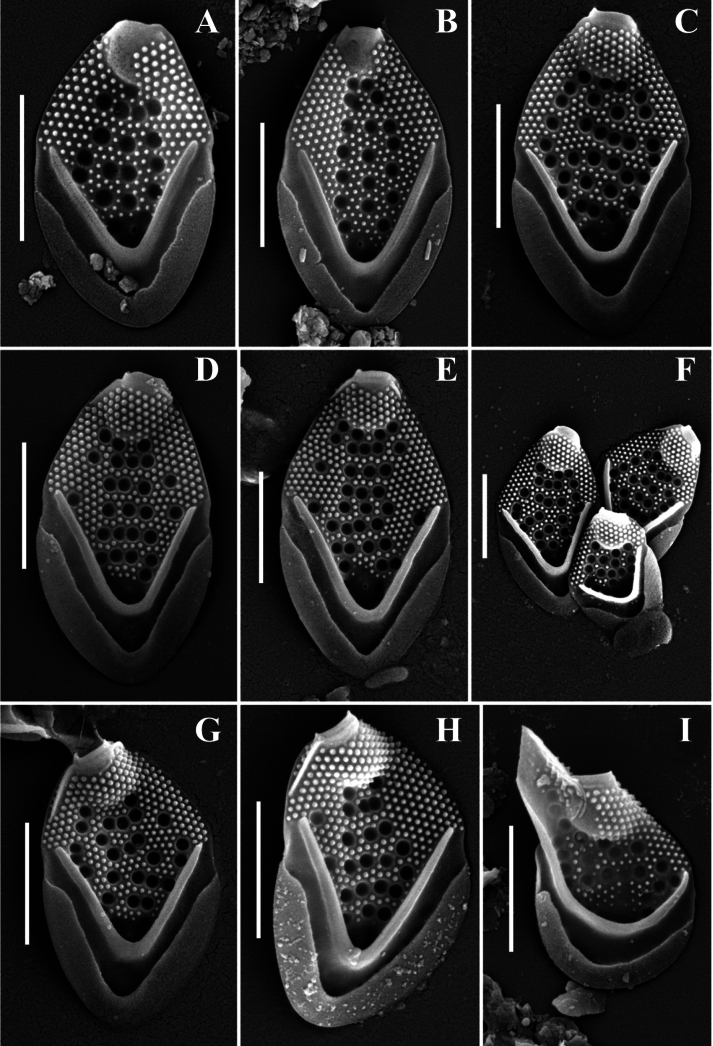
*Mallomonas monilifera* sp. nov. from environmental samples from Vietnam and Philippines, SEM. **A–E.** Body scales; **F.** Body and apical scales; **G–I.** Apical scales; **I.** Apical scale with wing-like projection. **A, B.**Mallomonas monilifera subsp. monilifera subsp. nov. Samples from Philippines; **C–I.**Mallomonas monilifera subsp. dongnaiensis subsp. nov. Samples from Vietnam. Scale bars: 2 µm.

The scales of Mallomonas monilifera subsp. monilifera (authentic strain C6/9) were more comparable to those of *M. guttata* in size (3.5–4.5 × 2.0–2.8 µm vs. 3.2–4.1 × 1.9–2.4 µm, respectively). The scales of *M. monilifera* recorded in natural samples from the Philippines (Fig. 8A, B) were also comparable in size to M. monilifera subsp. monilifera (3.8–4.5 × 2.5–2.8 µm) and can therefore be assigned to the type subspecies.

The scales of Mallomonas monilifera subsp. dongnaiensis were larger. The scales of strains 39_18, 32_18, and CT201 measured 4.1–5.0 × 2.2–2.9 µm. All these strains were isolated from water bodies in Cat Tien National Park (Vietnam). The study of preserved samples also confirmed the larger dimensions. For instance, the largest body scales of M. monilifera subsp. dongnaiensis were recorded in samples from Cat Tien National Park, measuring 4.5–5.6 × 2.7–3.3 µm. Thus, all recorded specimens of the *M. monilifera* morphotype in this region belong to the subspecies M. monilifera subsp. dongnaiensis (Fig. 8C–I).

The morphological differences between the isolates of *Mallomonas guttata* and *M. monilifera*, as well as the natural populations of *M. monilifera*, are summarized in Table 3.

The number of pits on the shield of the body scales also varied among different strains and fixed specimens. The revealed differences indicate that scale size and the number of pits on the shield surface cannot be used as reliable criteria for accurately differentiating between *Mallomonas monilifera* and *M. guttata*. The primary distinguishing feature, based on which *M. monilifera* can be readily distinguished from *M. guttata*, is the presence of a clearly visible rim around the pits on the shield, observable best in SEM images.

Studies of both species within the *Mallomonas guttata* complex have shown that scales with a significantly smaller number of pits can occur; these pits may be arranged in a row or scattered. Such scales are rare and are found on the same cell alongside standard scales with a larger number of pits. This fact raises the question of the validity of recognizing M. guttata var. simplex. However, based on our current data, we cannot yet draw a conclusion regarding the presence or synonymy of this taxon. M. guttata var. simplex was described from the temperate zone of North America. The pits on the shield of M. guttata var. simplex scales are more regularly arranged and larger (up to 0.3 µm according to the description). Additional comparative morphological and molecular studies of this variety are necessary.

Another aspect requiring discussion in relation to the study of *M. guttata* and *M. monilifera* cultures is the number of rimmed pores at the base of the V-rib. Our examination of cultures has shown that in old cultures, the formation of two or even more pores on the scales is possible. However, this has never been observed in material from fixed field samples. Therefore, we consider the presence of more than one rimmed pore to be an artifact of cultivation.

According to published data, *Mallomonas monilifera* has been previously found in China (see fig. 38, p. 195 in Kristiansen and Tong (1989); see figs 22, 23, p. 176 in Wei and Yuan (2001)) and Vietnam (see figs 23–25, p. 354 in Gusev et al. (2017)).

Thus, it can be concluded that *Mallomonas guttata* represents a complex of closely related taxa. The case of *M. monilifera* with its two genetic lineages, which are still poorly differentiated morphologically, illustrates the situation of evolutionarily young but separate lineages, as reported for *M. intermedia* Kisselev and *M. retimedia* Škaloud & al. (Škaloud et al. 2025a) and *Synura sphagnicola* (Korshikov) Korshikov and *S. rubra* Škaloud, Škaloudová & Jadrná (Škaloud et al. 2023). The number of putative species within the *M. guttata* group is not limited to the taxa described in this work. Various morphotypes attributed to *M. guttatasensu lato*, but differing in a number of important ultrastructural characteristics, have been previously discovered in Vietnam (Gusev and Nguyen 2011; Gusev 2013; Gusev et al. 2017, 2019, 2021, 2022b; Doan-Nhu et al. 2021; Gusev and Martynenko 2022). Observed variations include a significantly higher number of pits than indicated for *M. guttatasensu stricto*, pits arranged in transverse or longitudinal rows, pits located along the V-rib, and differences in scale size. This indicates that research on this variable group should be continued, but it must be done using combined morphological and molecular approaches to define the boundaries of morphological variability and the significance of characters. Drawing correct conclusions based solely on morphological data is no longer feasible.

## Supplementary Material

XML Treatment for Mallomonas guttata

XML Treatment for Mallomonas monilifera

XML Treatment for Mallomonas monilifera subsp. monilifera

XML Treatment for Mallomonas monilifera subsp. dongnaiensis

## ﻿Additional information

### Use of AI

No use of AI was reported.

### Author ORCIDs

Evgeniy S. Gusev https://orcid.org/0000-0002-7397-5808

Marina E. Ignatenko https://orcid.org/0000-0002-4451-7816

Nikita A. Martynenko https://orcid.org/0000-0002-7286-003X

Hoan Tran https://orcid.org/0000-0002-0143-9918

Chris Rey Lituanas https://orcid.org/0000-0002-8584-3757

Yulia A. Podunay https://orcid.org/0000-0002-0519-2908

### Data availability

All of the data that support the findings of this study are available in the main text.
